# The value of high-flow nasal cannula oxygen therapy after extubation in patients with acute respiratory failure

**DOI:** 10.6061/clinics/2017(09)07

**Published:** 2017-09

**Authors:** Hong-Zhuan Song, Juan-Xian Gu, Hui-Qing Xiu, Wei Cui, Gen-Sheng Zhang

**Affiliations:** I Department of Critical Care Medicine, Haining People’s Hospital, Zhejiang, China; II Department of Critical Care Medicine, Second Affiliated Hospital, Zhejiang University School of Medicine, Hangzhou, Zhejiang, China

**Keywords:** Oxygen Therapy, Acute Respiratory Failure, Extubation, Noninvasive Ventilation, High-Flow Nasal Cannula

## Abstract

**OBJECTIVE:**

To investigate the value of high-flow nasal cannula oxygen therapy after extubation in patients with acute respiratory failure.

**METHODS:**

A single-center, prospective, randomized, controlled pilot trial was conducted between January 2013 and December 2014. Sixty enrolled patients were randomized immediately after extubation into either a high-flow nasal cannula group (n=30) or an air entrainment mask group (n=30) at a fixed inspired oxygen fraction (40%). The success rate of oxygen therapy, respiratory and hemodynamic parameters and subjective discomfort (using a visual analogue scale) were assessed at 24h after extubation.

**RESULTS:**

The two groups were comparable at extubation. A total of 46 patients were successfully treated including 27 patients in the high-flow nasal cannula group and 19 patients in the air entrainment mask group. Compared to the air entrainment mask group, the success rate of oxygen therapy and the partial pressure of arterial oxygen were significantly higher and the respiratory rate was lower in the high-flow nasal cannula group. In addition, less discomfort related to interface displacement and airway dryness was observed in the high-flow nasal cannula group than in the air entrainment mask group.

**CONCLUSIONS:**

At a fixed inspired oxygen fraction, the application of a high-flow nasal cannula after extubation achieves a higher success rate of oxygen therapy and less discomfort at 24h than an air entrainment mask in patients with acute respiratory failure.

## INTRODUCTION

Acute respiratory failure (ARF) is the most common cause of admission to the intensive care unit (ICU) and often requires endotracheal intubation and mechanical ventilation 1. After improvements, these ventilated ARF patients should be considered for weaning and extubation. However, extubation remains a challenge in the critical care field, as a high reintubation rate exists (19%) 2. Patients are still pathophysiologically unstable after extubation, with symptoms such as incomplete recovery of primary diseases, oxygen deficit, upper airway obstruction, excess respiratory secretions, inadequate cough, or respiratory muscle weakness 3. Oxygen therapy is crucial to maintain the oxygen demand and to prevent the recurrence of ARF. A variety of conventional oxygen therapy (COT) systems that are classified as either fixed or variable performance are available, with various limitations 4. The air entrainment mask, as a fixed type, can increase dryness of the respiratory tract, influence daily patient activity, and cause patient discomfort. In addition, a nasal cannula cannot provide a constant oxygen concentration. Meanwhile, noninvasive ventilation (NIV) might not be suitable for all extubated patients due to their poor tolerance and cooperation 5.

Recently, the high-flow nasal cannula (HFNC) oxygen system (Optiflow, Fisher & Paykel Healthcare, Auckland, New Zealand), delivering heated and humidified high-flow (up to 60 L/min) gas, was introduced and has become widely used in clinical practice 6. It has several advantages, such as efficient humidification and airway mucociliary clearance, accurate fraction of inspired oxygen (FiO_2_) delivery and a low-level positive airway pressure 7. The concept of HFNC initially originated from the treatment of preterm infants as an alternative to nasal continuous positive airway pressure (CPAP) 8. The feasibility, efficacy, and tolerance of the HFNC have been tested in neonatal and pediatric care 9. More recently, the HFNC has been widely used in adults with hypoxemic ARF of different etiologies 10, in do-not-intubate patients 11, after cardiac surgery 12, in invasive practices such as bronchoscopy 13 and in other applications. Compared to COT, the HFNC can enhance patient comfort and tolerance, improve oxygenation, and reduce the reintubation rate 14,15. However, in some cases, the HFNC did not improve oxygenation or reduce the need for escalation of respiratory support 16. Thus, the exact efficacy of HFNC is still controversial or uncertain. Two recent randomized clinical trials have shown that use of an HFNC after extubation can decrease the need for reintubation compared with COT 15 and is not inferior to NIV for preventing reintubation and postextubation respiratory failure 17. However, in these two studies, the FiO_2_ was adjustable, and the studies included patients who were at either low risk 15 or high risk for reintubation 17.

Given that the HFNC has the intrinsic advantages of efficient humidification and dynamic positive airway pressure 7, we hypothesized that at a fixed FiO_2_ and use of an HFNC after extubation could achieve a higher success rate of oxygen therapy than an air entrainment mask in ARF patients.

## MATERIALS AND METHODS

This study was approved by the Ethics Committee of Haining People’s Hospital, Zhejiang, China. Written informed consent was obtained from included patients or their surrogates. Mechanically ventilated ARF patients who were admitted to the 24-bed adult comprehensive ICU at Haining People’s Hospital between January 2013 and December 2014 were prospectively included. The criteria for ARF were based on the conventional definition: partial pressure of arterial oxygen (PaO_2_) <60 mmHg, partial pressure of arterial carbon dioxide (PaCO_2_) >45 mmHg, or both 18. Patients were included in this study if they had undergone mechanical ventilation for at least 48h and were ready for tracheal extubation after clinical weaning assessments, according to the international consensus conference on weaning 19; the criteria for weaning assessments in this study included evidence of clinical improvement of the original pathologic process leading to ARF, relative cardiovascular stability with (at most) a minimum requirement for vasopressors, adequate mentation, efforts at spontaneous ventilation, and adequate oxygenation (defined as a PaO_2_/FiO_2_ of at least 150 mmHg with FiO_2_≤0.4 and PEEP≤8 cmH_2_O). Patients were excluded from this study if they had poor cooperation, a tracheostomy, or a decreased level of consciousness (Glasgow Coma Scale score of 12 points or less); were younger than 18 years old or pregnant; or did not sign the informed consent form.

The patients who met the weaning criteria and successfully passed the spontaneous breathing trial with 7 cmH_2_O of pressure support for 30 to 120 min were eligible and ready for tracheal extubation. The baseline time point was defined as the end of the spontaneous breathing test and immediately before extubation. Immediately after extubation, the patients were randomized into two groups: oxygen treatment by HFNC (PT101AZ, Fisher & Paykel Healthcare, Auckland, New Zealand) or air entrainment mask (Jinlin Medical Appliances Factory, Hangzhou, China). Randomization was accomplished using a computer-generated random number sequence. In both groups, the FiO_2_ was set at 40%. The flow level of the HFNC began at 60 L/min and was adjusted downward in 5- to 10-L/min decrements as the target oxygenation improved or stabilized, while the flow rate of the air entrainment mask was set at 10 L/min. The target oxygenation in our study was a pulse oxygen saturation (SpO_2_) of 94–98% for most patients (hypoxic respiratory failure) or 88–92% for those with hypercapnic respiratory failure. Demographic data such as age, gender, and Acute Physiology and Chronic Health Evaluation II (APACHE-II) score were recorded at baseline. Electrocardiography variables, heart rate, blood pressure, mean arterial pressure, respiratory rate, SpO_2_, and arterial blood gas values were monitored at baseline and 24h after extubation. A visual analog scale 20 [from 0 (no discomfort) to 10 (maximum discomfort)] was used to assess patient discomfort related to airway dryness or the interface. In addition, adverse events, including replacement of the original oxygen system (shifting to an HFNC from the air entrainment mask in this study) and requiring NIV or endotracheal intubation, were also recorded. Decisions to apply NIV or endotracheal intubation were based on previous criteria 21,22. The ventilator mode of NIV was set to pressure support mode or pressure controlled mode. The criteria for shifting to an HFNC from the air entrainment mask were based on a previous study 23 and were modified as follows: respiratory rate >30 breaths/min, SpO_2_ <90%, or intolerance to the air entrainment mask. The primary objective of this study was to evaluate the success rate of oxygen therapy at 24h after extubation. In the present study, successful oxygen therapy was defined as not requiring a replacement oxygen device, NIV, or reintubation within 24h after extubation. The secondary objectives of the study were to investigate respiratory variables, hemodynamic variables, and patient discomfort at 24h after extubation.

The results of this study were presented as absolute numbers and percentages or means and standard deviations for continuous data if normally distributed and medians and ranges if not normally distributed. Comparisons between the two groups were performed using a *t* test or Mann-Whitney U test for metric data and the chi-square test for categorical data. A two-sided *p-*value of less than 0.05 was considered significant. IBM SPSS Statistics for Windows software (IBM Corp. Released 2011. Version 20.0. Armonk, NY, USA) was used for data analysis.

## RESULTS

**Figure 1:**
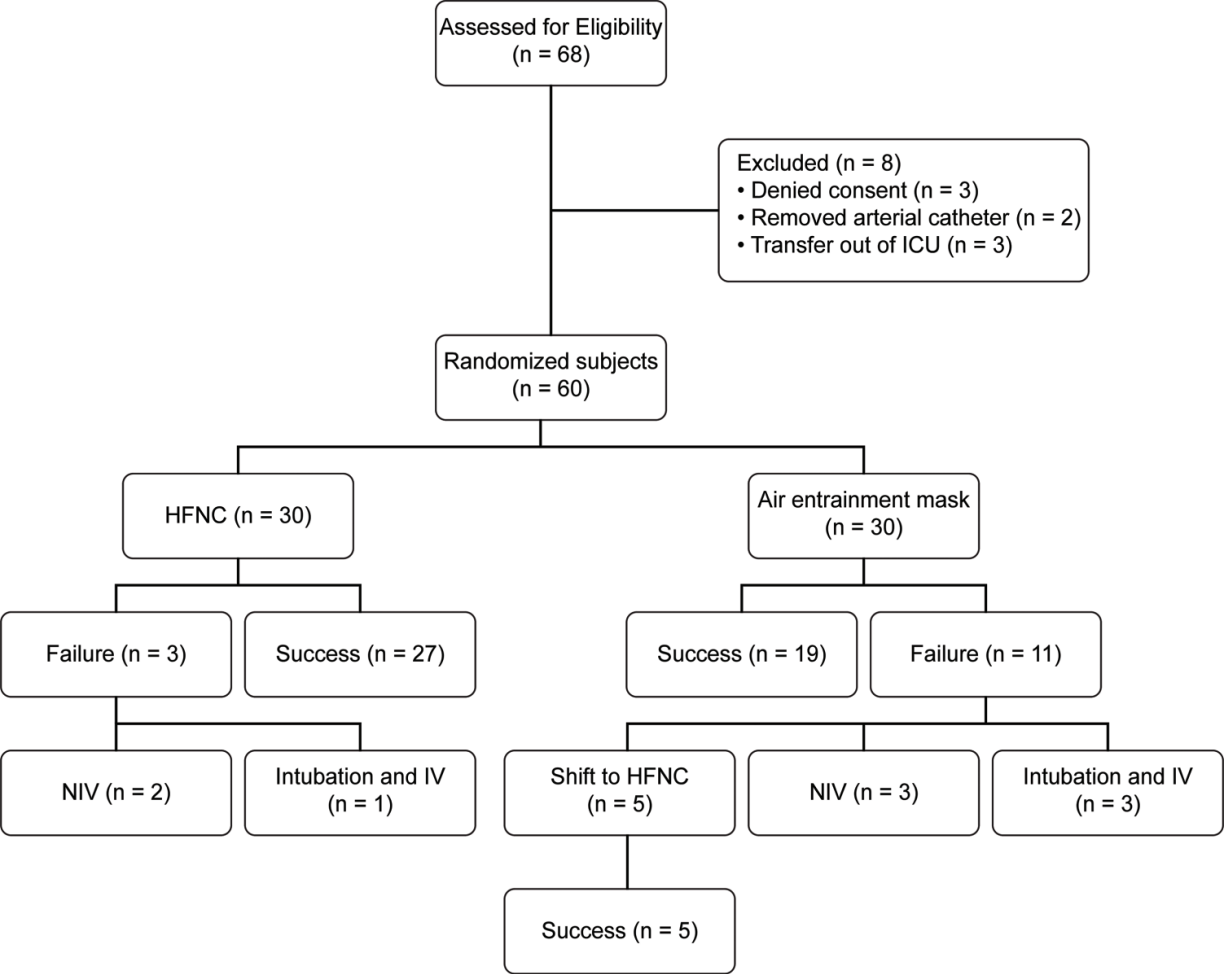
Flow diagram of participants enrolled in the study and associated clinical outcomes after extubation. ICU: Intensive care unit; HFNC: high-flow nasal cannula; NIV: noninvasive ventilation; IV: invasive ventilation.

Between January 2013 and December 2014, 68 patients met the inclusion criteria and were eligible to participate in this study (flow chart in Figure 1). Eight patients were excluded from the study: three patients declined informed consent, two patients removed invasive arterial monitoring catheters during the study period, and three other patients were transferred out of the ICU on the day of extubation. Eventually, 30 patients were included in the HFNC group, and 30 patients were included in the air entrainment mask group. The demographic and clinical characteristics of the two groups at baseline were comparable, as shown in Table 1.

Among the 60 patients, 46 were successfully treated by initial oxygen therapy within 24h after extubation, including 27 in the HFNC group and 19 in the air entrainment mask group. The success rate of oxygen therapy by HFNC (27/30, 90%) was significantly higher than that by the air entrainment mask (19/30, 63.3%) (*p*=0.01) (Table 2). Of the 11 patients who failed treatment with the air entrainment mask, 3 received NIV, 3 required reintubation, and 5 were shifted to the HFNC (these patients were all successfully treated by HFNC). Although no significant difference was found, the rate of ventilator support (3/30, 10%) in the HFNC group was lower than that in the air entrainment mask group (6/30, 20%). A total of four patients required reintubation: one in the HFNC group (1/30, 3.33%) and three in the air entrainment mask group (3/30, 10%); the difference was not statistically significant (Table 2).

At 24 h after extubation, the average flow rate in the HFNC group was 36.8±2.8 L/min. In addition, the HFNC significantly improved the PaO_2_ and SpO_2_ at 24h after extubation compared to the air entrainment mask (83.2±10.5 mmHg *vs*. 74.5±13.1 mmHg, *p*=0.016; 98.0±1.3% *vs*. 96.9±1.4%, *p*=0.011, respectively). The PaCO_2_ values were similar between the two groups (*p*=0.591, Table 2). The respiratory rate was significantly lower for the HFNC group than for the air entrainment mask group (22±3.6 breaths/min *vs*. 26±4.3 breaths/min, *p*=0.003). No significant differences in heart rate and mean arterial pressure were observed between the two groups (Table 2).

**Figure 2:**
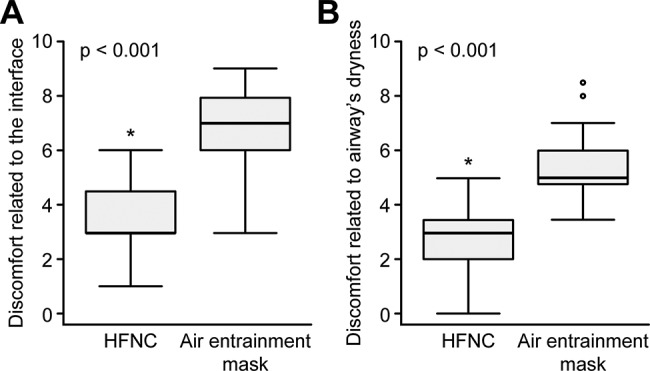
High-flow nasal cannula (HFNC) oxygen therapy improved the discomforts related to the interface and symptoms of airway dryness compared to the air entrainment mask. Patients’ discomforts were assessed by scores related to the interface (panel A) or airway dryness (panel B) via a visual analog scale [from 0 (no discomfort) to 10 (maximum discomfort)] (*p<0.001 compared to the air entrainment mask).

Discomforts related to the interface and symptoms of airway dryness were significantly lower in the HFNC group than those in the air entrainment mask group [3(3-4.5) *vs*. 7(6-8); 3(2-3.5) *vs*. 5(4.7-6), both *p*<0.001] (Figure 2).

## DISCUSSION

The main findings of this study were as follows: after extubation of mechanically ventilated ARF patients, the application of an HFNC achieved a higher success rate of oxygen therapy within 24 h than the air entrainment mask. In addition, use of the HFNC resulted in improved oxygenation, a decreased respiratory rate, and reduced patient discomfort compared to the air entrainment mask. After failure with the air entrainment mask, five patients were shifted to the HFNC group and ultimately achieved success with oxygen therapy, thus avoiding the introduction of NIV or reintubation. Consistent with other studies 14,15, our study also confirmed that the HFNC is a promising oxygen therapy device for patients with ARF after extubation. Moreover, this procedure could even be used as an alternative after the failure of other oxygen devices to reduce the need for NIV or reintubation; these findings indirectly supported the conclusion obtained by Hernández et al. 17, namely, that HFNC was not inferior to NIV in terms of reintubation or postextubation failure.

The HFNC has been increasingly used in patients after extubation, although its efficacy remains unclear. Tiruvoipati et al. 24 showed that the HFNC was better tolerated than a high-flow face mask in 50 extubated patients, despite a similar effectiveness of oxygen delivery. In 2014, Rittayamai et al. 25 found that the HFNC improved dyspnea and the respiratory and heart rates compared to a non-rebreathing mask in 17 extubated patients. However, these two studies observed only the short-term efficacy of the HFNC 24,25. The relative long-term efficacy of the HFNC in extubated patients was investigated in two recent well-designed studies 14,15. Maggiore et al. 14 found that HFNC oxygen therapy resulted in significantly better oxygenation, better comfort, and a lower reintubation rate (3.8%) during the 48-h study period than COT in 105 critically ill patients. Similarly, Hernández et al. 15 also showed that HFNC application resulted in a significantly lower reintubation rate (4.9%) than COT (12.2%) within 72 h in 527 adult patients at low risk for reintubation. However, the influence of the FiO_2_ on the efficacy of the HFNC for oxygen therapy was not excluded in these two studies. Our study extended the application of the HFNC to use in patients after extubation, even for those with a fixed FiO_2_.

The exact mechanism of the beneficial effects observed from the application of the HFNC remains unclear. The following factors should be considered. (a) The high-flow oxygen (up to 60 L/min) delivered by the HFNC meets or exceeds the patient’s peak inspiratory demand, which might deliver a more accurate FiO_2_ with a fixed FiO_2_. (b) The delivery of high-flow oxygen flushes the anatomical dead space of the upper airway, creating a reservoir of fresh gas available for every breath to minimize re-breathing of CO_2_, which would improve the efficiency of ventilation and oxygen delivery. The PaCO_2_ level at 24 h after oxygen therapy was similar between the two groups, which suggested that the HFNC had no significant effect on the PaCO_2_ values in our study. (c) In contrast to COT, the HFNC system can generate positive airway pressure to increase airway compliance and reduce the breathing work 26. (d) The sufficiently heated humidified air produced by the HFNC facilitates secretion clearance and decreases bronchial hyper-response symptoms 27. (e) Better subjective comfort generally results in better subject compliance and a better outcome 28. Our study confirmed that HFNC oxygen therapy led to reduced patient discomfort compared to the air entrainment mask. We also showed that the rate of ventilatory support (NIV or reintubation) in the air entrainment mask group tended to be higher than that in the HFNC group, and this trend might be associated with the mismatch between the oxygen flow and the patient’s inspiratory demand, the patients’ intolerance to the mask, the instability of the delivered FiO_2_, and/or insufficiency of heating and humidification 4,7. An oxygen target (a SpO_2_ of 94–98% for most patients) and a fixed FiO_2_ of 40% were set in the present study, which could raise the question of how to improve oxygenation. Indeed, we performed other measures, such as maintenance of a high flow rate and/or sputum removal; NIV and reintubation were also considered if necessary. Most patients in the current study met the target oxygenation at a fixed FiO_2_ of 40%, and only 6.67% (4/60) required reintubation, mainly due to respiratory muscle weakness, cardiac failure, excess respiratory secretions, and/or changes in mental status.

Our study has several limitations. (a) The small number of patients and the pilot study design resulted in a relatively low statistical power. Based on a superior design according to the acute sample size using SAS software (Cary, North Carolina USA), the sample size of 30 in each group would have a power of 0.807, with a level of significance of 0.05 (the success rates of oxygen therapy were 90% and 63% in the HFNC and air entrainment mask groups, respectively) (Table 2). (b) In this study, we considered the 5 patients with successful shifts to the HFNC oxygen treatment as treatment failures to their initial oxygen therapy by air entrainment mask (Figure 1). To some extent, it might be reasonable to crossover these 5 patients because this finding further suggests that HFNC oxygen treatment might be superior to use of an air entrainment mask. As this study was open label by nature, it was difficult to blind participants and clinicians to the allocated oxygen therapy, which is a problem inherent to many studies of medical devices. This problem was relatively weakened by the randomization techniques and the comparability of the groups at baseline in the current study. (c) The APACHE-II scores in both groups were approximately 12; thus, the severity of the population in this study was relatively minor. Whether HFNC therapy is suitable for more serious patients with respiratory failure after extubation requires further investigation. (d) Although a fixed FiO_2_ setting was used in both groups in our study, the actual FiO_2_ delivered to the patient might be inconsistent with the set FiO_2_ in some cases. For example, the actual FiO_2_ delivered to the patient could be lower than 40% with both the air entrainment mask (in case of a higher respiratory rate or tidal volume) and the HFNC (when lower flow rates were used while the patient’s peak inspiratory flow was higher). The use of greater flow rates with the HFNC could allow more stable delivery of the FiO_2_. Unfortunately, we did not measure the actual FiO_2_ delivered to the patient. (e) The potential beneficial effects of the HFNC on sputum clearance and bronchial hyper-response symptoms were not assessed, which might also contribute to the higher success rate of initial oxygen treatment by improving mucociliary function and reducing the work of breathing. (f) The assessment of patient discomfort was subjective. However, this method is widely used in the measurement of breathlessness and other symptoms 20. (g) Indeed, we also clinically titrated the amount of oxygen according to the patients’ needs, but not with a fixed FiO_2_. However, the aim of the current study was to investigate whether the potential mechanisms underlying the benefit of HFNC oxygen treatment were unrelated to the amount of oxygen, and we used a fixed FiO_2_.

In summary, HFNC oxygen therapy after extubation in mechanically ventilated ARF patients can achieve a higher success rate of oxygen therapy, improved oxygenation, and a lower occurrence of discomfort than an air entrainment mask. Thus, HFNC oxygen therapy might be a promising treatment for ARF patients after extubation.

## AUTHOR CONTRIBUTIONS

Song HZ, Gu JX and Zhang GS designed the study. Song HZ, Gu JX and Xiu HQ were responsible for the patient care, technical advice, data acquisition, statistical analysis, interpretation of the data and manuscript writing. Zhang GS and Cui W revised the manuscript. All authors read and approved the final version of the manuscript.

## Figures and Tables

**Table 1:** Baseline patient characteristics.

	HFNC (n=30)	Air entrainment mask (n=30)
Male, n (%)	116 (53.3)	18 (60)
Age, years	66±14	71±13
APACHE-II	12.87±3.0	12.36±3.29
Causes of ARF, n (%)		
Pneumonia	12 (40)	13 (43.3)
COPD exacerbation	7 (23.3)	6 (20)
Cardiogenic pulmonary edema	5 (16.7)	6 (20)
Multiple trauma	1 (3.3)	2 (6.7)
Cardiac arrest	2 (6.7)	1 (3.3)
Others*	3 (10)	2 (6.7)
Length of MV before inclusion (days)	5.5±3.4	5.4±2.8
Heart rate (beats/min)	82.80±9.85	81.53±8.92
Mean arterial pressure (mmHg)	83±9	83±10
PaCO_2_ (mmHg)	41.5±6.7	42.3±7.1
PaO_2_ (mmHg)	82.8±11.0	81.7±11.6
SpO_2_ (%)	96.2±2.3	95.1±2.9

* Others included psychiatric drug poisoning (n=1), severe acidosis (n=3), and epileptic seizures (n=1).

HFNC: High-flow nasal cannula; APACHE-II: Acute Physiology and Chronic Health Evaluation II score; ARF: Acute respiratory failure; COPD: Chronic obstructive pulmonary disease; MV: Mechanical ventilation.

**Table 2:** Primary and secondary outcomes in the two treatment groups.

	HFNC	Air entrainment mask	*p*
Primary outcomes	n=30	n=30	
Success rate of oxygen therapy	27/30 (90%)	19/30 (63.33%)	0.012
NIV	2 (6.67%)	3 (10%)	0.639
Endotracheal intubation	1 (3.33%)	3 (10%)	0.290
Replacement of oxygen device	0 (0%)	5 (16.67%)	0.062
Secondary outcomes	n=27	n=19	
PaO_2_ (mmHg)	83.2±10.5	74.5±13.1	0.016
SpO_2_ (%)	98.0±1.3	96.9±1.4	0.011
PaCO_2_ (mmHg)	41.4±6.5	42.2±13.1	0.591
Respiratory rate (breaths/min)	22±4	26±4	0.003
Heart rate (beats/min)	85±9	87±14	0.598
Mean arterial pressure (mmHg)	85±8	86±9	0.824

HFNC: High-flow nasal cannula; NIV: Noninvasive ventilation.
